# Positive mental health among health professionals working at a psychiatric hospital

**DOI:** 10.1371/journal.pone.0178359

**Published:** 2017-06-07

**Authors:** Louisa Picco, Qi Yuan, Janhavi Ajit Vaingankar, Sherilyn Chang, Edimansyah Abdin, Hong Choon Chua, Siow Ann Chong, Mythily Subramaniam

**Affiliations:** 1 Research Division, Institute of Mental Health, Singapore, Singapore; 2 Chief Executive Office, Institute of Mental Health, Singapore, Singapore; University of Campania "L. Vanvitelli", ITALY

## Abstract

**Background:**

Positive mental health (PMH) is a combination of emotional, psychological and social well-being that is necessary for an individual to be mentally healthy. The current study aims to examine the socio-demographic differences of PMH among mental health professionals and to explore the association between job satisfaction and total PMH.

**Methods:**

Doctors, nurses and allied health staff (n = 462) completed the online survey which included the multidimensional 47-item PMH instrument as well as a single item job satisfaction question. Associations of PMH with job satisfaction were investigated via linear regression models.

**Results:**

Significant differences in PMH total and domain specific scores were observed across socio-demographic characteristics. Age and ethnicity were significantly correlated with PMH total scores as well as various domain scores, while gender, marital and residency status and the staff’s position were only significantly correlated with domain specific scores. Job satisfaction was also found to be a significantly associated with total PMH.

**Conclusion:**

The workplace is a key environment that affects the mental health and well-being of working adults. In order to promote and foster PMH, workplaces need to consider the importance of psychosocial well-being and the wellness of staff whilst providing an environment that supports and maintains overall health and work efficiency.

## Introduction

Positive aspects of mental health comprise concepts such as well-being and happiness. An increased emphasis relating to this construct has seen a global shift which acknowledges the need to address mental health as an integral part of improving overall health and well-being [1,2]. This is reflected in the World Health Organization’s (WHO) definition of health which states ‘health is a state of complete physical, mental and social well-being and not merely the absence of disease or infirmity’. Similarly, Prince et al [3] describes the concept of there being ‘no health without mental health’, where they describe the connectedness and interactions between mental and physical conditions as protean, with one being able to affect the other and vice versa. More specifically, the WHO defines mental health as ‘a state of well-being in which every individual realizes his or her own potential, can cope with the normal stresses of life, can work productively and fruitfully, and is able to make a contribution to her or his community’ [1,2], a definition which encompasses an individualistic and societal perspective. Accordingly, mental health and mental illness are related, yet are separate and distinct phenomena.

Well-being is a complex construct which is commonly derived and operationalized from two general perspectives: hedonic well-being and eudaimonic well-being [4,5]. Hedonic or subjective well-being refers to emotional aspects including happiness, satisfaction and interest with life, while eudaimonic or psychological well-being focuses on human functioning and achievement of self-realization. Experts have now integrated theories and components of hedonic and eudaimonic well-being into composite models of mental health [4]. Research has shown that mental health and well-being are influential to various outcomes and aspects of life including an individual’s lifestyle and behaviour [6], their social performance and interpersonal relationships [7], as well as contributing to improved quality of life [8], and successful ageing [9].

The concept and interpretation of mental health and more specifically positive mental health (PMH) however is not unanimous as it can vary in terms of place, culture and context [10]. Broadly speaking, PMH is a combination of emotional, psychological and social well-being that is necessary for an individual to be considered mentally healthy [11]. An earlier study that qualitatively explored the concept of PMH in Singapore, revealed that PMH encompassed various concepts and was defined as the ‘ability to build and maintain relationships, to have active coping and interpersonal skills, provide and receive emotional support, pursue personal growth and autonomy, and participate in religious and spiritual practices’ [12].

Singapore is a highly urbanized country in Southeast Asia, with a strong globalized economy and a population of 5.5 million [13]. Under the first National Mental Health Policy and Blueprint, which was launched in 2007, the importance of promoting mental well-being and building resilience was emphasized and as a result, an instrument was developed to measure PMH amongst the local multi-ethnic population of Singapore [12,14]. The PMH instrument consists of six domains, covering both psychological and subjective aspects of well-being. It has been validated in the local population [14] and among people with mental illness [15] and has also been used in caregiver populations, however less is known about PMH amongst mental health professionals.

More recently the PMH movement has extended beyond clinical setting and has found importance in workplace settings, where it has been found that by promoting mental health among employees can result in improvements in organizational health [16]. Studies have found that employees with low levels of well-being are more likely to leave their organisation as a result of job dissatisfaction [17], while positive correlations between well-being and performance [18] have also been observed. More specifically, research has shown for nurses and allied health professionals, promoting well-being strengthens work commitment and performance [19,20].

The phenomenon of burnout among mental health professionals is also of increased interest, with findings consistently showing high burnout amongst this group [21]. Higher burnout is also commonly reported among psychiatric nurses compared to nurses of other specialties [22]. In a recent local study among mental health professionals working at the Institute of Mental Health (IMH), Yang et al [23] similarly found staff were experiencing high levels of stress and burnout. Burnout has been shown to be associated with lower job satisfaction, decreased quality of care [24,25], high staff turnover, decreased patient satisfaction and diminished work efficiency [26,27]. Mental illnesses have also been shown to be higher among health professionals. Letvak et al., [28] reported a twice as high prevalence of depression among nurses compared to the adult population in USA.

The aims of the current study were to explore and examine the socio-demographic differences and identify correlates for domain specific and total PMH among a multi-ethnic group of mental health professionals working at IMH. Secondly, the study aims to explore the association between job satisfaction and total PMH. We hypothesize that increased job satisfaction will be associated with higher PMH among mental health professionals.

## Material and methods

### Participants and procedure

Doctors, nurses, psychologists, pharmacists, occupational therapists, physiotherapists, case managers and medical social workers working at IMH, the sole tertiary psychiatric hospital in Singapore, were invited to participate in the online survey, which was administered via the survey application ‘Questionpro’. Staff were informed of the study via email and were sent a link to the online survey which was only sent to doctors, nurses and allied health staff, using relevant hospital group emails lists. The email outlined the inclusion criteria which required respondents to be: (i) Singapore citizens, Permanent Residents or work permit holders; (ii) doctors, nurses, psychologists, pharmacists, occupational therapists, physiotherapists, case managers or medical social workers currently working at IMH; (iii) aged 21 years and above and; (iv) able to complete the online survey in English. Staff who were willing to participate in the survey were required to complete a screener section to assess their eligibility and were then instructed to read an online consent form, and by clicking on the ‘agree’ link, this indicated their willingness and informed consent to participate in the study.

As the current study was part of a larger study exploring associative stigma among staff at IMH, it was estimated that a sample size of approximately 200 nurses and 200 allied health staff would be needed to explore differences in associative stigma amongst the two groups, where sample size calculations were performed using PS (power and sample size calculation) software for comparing means. Accordingly, once this limit had been reached, subsequent staff who wished to participate in the survey were sent a message informing them recruitment had ceased. In total 470 participants completed the study, with eight cases being removed due to unreliable data or staff not meeting the inclusion criteria. Inclusion criteria were also evaluated when staff came to collect their inconvenience fee. They were instructed to bring along their staff card and national registration identity card, which were used to confirm respondent’s position and department and age, respectively. On average, the online survey took approximately 10–15 minutes to complete and data collected was downloaded from Questionpro in SPSS’s data file format. Ethical approval was obtained from the Domain Specific Review Board of the National Healthcare Group, Singapore prior to the launch of the survey.

### Measures

The 47-item PMH instrument consists of six domains of mental health; general coping (GC, 9 items), emotional support (ES, 7 items), spirituality (7 items), interpersonal skills (IS, 9 items), personal growth and autonomy (PGA, 10 items) and global affect (GA, 5 items) [14]. All items are positively worded and include statements such as ‘I try not to let it bother me’, ‘I try to get emotional support from family and friends’ and ‘I have confidence in the decisions I make’. For the first five domains, respondents were asked to indicate how much each item describes them on a rating scale from ‘not at all like me (1)’ to ‘exactly like me (6)’. The GA domain required staff to indicate how often over the past four weeks they felt calm, happy, peaceful, relaxed and enthusiastic on a 5-point response scale. PMH total and domain specific scores are obtained by summing scores of the respective items and dividing by the number of items in each domain, where higher scores indicate higher PMH. The instrument included ten additional negatively worded items that served as ‘filler items’ to check pattern responses and did not contribute to the scores.

The PMH instrument has been locally tested for construct validity, differential item functioning, reliability and criterion validity amongst the general population [14]. More specifically, the results of goodness-of-fit indices fit the data well (Comparative fit indexes (CFI) = 0.95–0.96, Tucker-Lewis Index (TLI) = 0.95–0.96, Root Mean Square Error Approximation (RMSEA) = 0.05–0.06). The Cronbach’s alpha coefficient for the PMH total score was 0.96. The alpha coefficients for GC, PGA, Spirituality, IS, ES and GA scores were 0.89, 0.93, 0.94, 0.89, 0.89, and 0.89 respectively. Among the current sample, the PMH instrument also showed good internal consistency (Cronbach's α = 0.96) and the Cronbach's alpha was also high for each of the domains: GC = 0.91, PGA = 0.95, Spirituality = 0.96, IS = 0.87, ES = 0.89, GA = 0.90.

Socio-demographic information was captured including age, gender, residency status, ethnicity, marital status, education and employment related information (current position and years of employment at IMH). Staff were also asked to rate their overall job satisfaction on a 10-point scale, where 1 indicated they are very dissatisfied and 10 indicated they were very satisfied.

### Statistical analysis

Descriptive analyses were performed for socio-demographic and dependent variables. Continuous variables were presented as mean and standard deviation (SD) and frequencies and percentages were calculated for all other categorical variables. Independent t-tests and one-way ANOVA with Bonferroni post-hoc tests were performed to establish the differences for the PMH total and domain specific scores across different socio-demographic subgroups. Independent t-tests were used when the socio-demographic variables only have two categories (e.g. gender and marital status), whereas one-way ANOVA with Bonferroni post-hoc tests were used when the socio-demographic variables have more than two categories (e.g. ethnicity and education level). Multivariate linear regression analysis was used to explore the correlations between socio-demographic variables and the PMH total and domain specific scores after controlling for confounding. In order to explore the relationship between job satisfaction and PMH total scores, a series of linear regressions were performed. In the first step, job satisfaction was entered as a quantitative independent variable to predict the PMH total scores in the unadjusted model. The adjusted models were then analysed by including additional factors in the following sequence: socio-demographic characteristics (i.e. age, gender, ethnicity, marital status, educational level, residency status, position held by the staff, and number of years the staff had worked at IMH) in model 1; and both socio-demographics and ‘whether the participant had family or close friends diagnosed with a mental illness’ in model 2. A two-sided p-value of 0.05 was considered as statistically significant. All data analyses were conducted using SAS 9.3.

## Results

The distribution of socio-demographic characteristics is presented in Table 1. The sample (n = 462) comprised 58 doctors, 201 nurses and 203 allied health staff. Allied health staff comprised case managers (n = 57), medical social workers (n = 47), occupational therapists (n = 25), pharmacists (n = 28), physiotherapists (n = 3) and psychologists (n = 43) (See S1 Table for PMH total and domain specific scores across allied health disciplines). The majority of the sample were female (63%), Chinese (60.2%), and had been working at IMH for between one and five years (42.2%). The mean PMH total score was 4.54 while the mean domain specific scores ranged from 4.02 (for spirituality) to 4.89 (for ES). Job satisfaction scores ranged from 1 to 10, with the mean score being 7.16 (Table 2).

**Table 1:** Characteristics of the study sample.

Characteristics		n	%
**Age (mean years, SD)**		36.4	10.6
**Gender**	Female	291	63
	Male	171	37
**Ethnicity**	Chinese	278	60.2
	Malay	36	7.8
	Indian	64	13.8
	Filipino	59	12.8
	Burmese	16	3.5
	Others	9	1.9
**Marital status**	Never married	205	44.4
	Ever married	257	55.6
**Education level**	Secondary/ ITE/'O' level	18	3.9
	'A' level/diploma	49	10.6
	Bachelor	241	52.2
	Master or above	154	33.3
**Residential status**	Singapore Citizen	320	69.2
	Permanent Resident	59	12.8
	Non Resident	83	18.0
**Position held**	Doctor	58	12.6
	Nurse	201	43.5
	Allied Health	203	43.9
**Years in IMH**	Less than 1 year	52	11.3
	1–5 years	195	42.2
	6–10 years	103	22.3
	More than 10 years	112	24.2

**Table 2:** Descriptive statistics for PMH total and domain specific scores and job satisfaction scores.

	Mean	SD	Min	Max
**PMH Instrument**				
Total Positive Mental Health	4.54	0.69	2.89	6.00
General Coping	4.54	0.84	2.00	6.00
Emotional Support	4.89	0.83	1.71	6.00
Spirituality	4.02	1.58	1.00	6.00
Personal Growth and Autonomy	4.60	0.84	2.20	6.00
Interpersonal Skills	4.65	0.71	2.56	6.00
Global Affect	4.61	0.84	2.00	6.00
**Job Satisfaction**	7.16	1.56	1.00	10.00

Table 3 shows the mean (SD) of PMH total and domain specific scores among the sample and by socio-demographic characteristics. Bivariate analysis revealed there were significant differences particularly for ethnicity, residency status, position held by the staff and number of years of employment at IMH. After adjusting with Bonferroni’s post hoc tests, more specific differences were identified. For ethnicity, significant differences were observed for PMH total and all the domain specific scores, with the most consistent finding being that Filipinos had higher scores across all aspects compared to Chinese. For residency status, differences were seen for PMH total scores (p<0.001) as well as GC (p = 0.004), PGA (p<0.001), IS (p<0.001) and GA (p = 0.002), with citizens scoring lower than non residents on all aspects. With regards to employment related demographics, nurses scored higher than allied health staff for GC, PGA, IS and GA (p<0.001) and higher than allied health staff and doctors for total PMH (p = 0.003) and spirituality (p<0.001). Lastly, when compared to staff working at IMH for 6–10 years, those working at the hospital for more than 10 years had higher PMH total (p = 0.001), spirituality (p = 0.006) and GA (p = 0.007) scores and higher PMH total (p = 0.049) and PGA (p = 0.018) scores compared to those employed 1–5 years.

**Table 3:** PMH total and domain scores across socio-demographic characteristics.

	Total PMH Score		General Coping		Emotional Support		Spirituality		Personal Growth and Autonomy		Interpersonal Skills		Global Affect	
	Mean ± SD	*P*	Mean ± SD	*P*	Mean ± SD	*P*	Mean ± SD	*P*	Mean ± SD	*P*	Mean ± SD	*P*	Mean ± SD	*P*
**Overall Sample**	4.54 ± 0.69		4.54 ± 0.84		4.89 ± 0.83		4.02 ± 1.58		4.60 ± 0.84		4.65 ± 0.71		4.61 ± 0.84	
**Gender**														
Male	4.52 ± 0.64	0.6	4.60 ± 0.70	0.188	4.73 ± 0.77	0.003	3.91 ± 1.62	0.238	4.65 ± 0.77	0.317	4.63 ± 0.69	0.644	4.64 ± 0.75	0.499
Female	4.56 ± 0.72		4.50 ± 0.91		4.98 ± 0.86		4.09 ± 1.56		4.57 ± 0.87		4.66 ± 0.73		4.59 ± 0.90	
**Ethnicity**														
Chinese	4.37 ± 0.64	<0.001^a^^b^^c^^d^	4.41 ± 0.80	<0.001^b^^c^	4.79 ± 0.82	0.001^b^	3.63 ± 1.57	<0.001^a^^b^^c^^d^^e^^f^	4.45 ± 0.82	<0.001^b^^g^	4.53 ± 0.66	<0.001^b^	4.49 ± 0.81	<0.001^b^^d^
Malay	4.82 ± 0.75		4.71 ± 0.96		5.13 ± 0.77		5.27 ± 0.76		4.64 ± 1.03		4.78 ± 0.82		4.72 ± 0.99	
Indian	4.63 ± 0.67		4.53 ± 0.84		4.87 ± 0.89		4.09 ± 1.56		4.83 ± 0.76		4.78 ± 0.74		4.64 ± 0.86	
Filipino	5.12 ± 0.59		5.05 ± 0.74		5.28 ± 0.68		5.11 ± 1.00		5.01 ± 0.68		4.99 ± 0.73		5.02 ± 0.72	
Burmese	4.81 ± 0.69		4.60 ± 0.94		4.64 ± 0.97		4.30 ± 1.40		4.82 ± 0.86		4.78 ± 0.62		5.07 ± 0.88	
Others	4.26 ± 0.56		4.69 ± 0.65		4.80 ± 0.97		2.86 ± 1.81		4.43 ± 0.77		4.51 ± 0.61		4.11 ± 0.63	
**Marital status**														
Never married	4.52 ± 0.72	0.5216	4.58 ± 0.87	0.438	4.88 ± 0.84	0.845	3.95 ± 1.61	0.399	4.56 ± 0.90	0.423	4.69 ± 0.73	0.384	4.54 ± 0.93	0.109
Ever married	4.56 ± 0.67		4.51 ± 0.82		4.89 ± 0.83		4.08 ± 1.56		4.63 ± 0.78		4.63 ± 0.70		4.67 ± 0.76	
**Education level**														
Secondary /ITE/ 'O' level	4.69 ± 0.81	0.065	4.77 ± 0.92	0.460	4.76 ± 1.09	0.695	4.95 ± 1.05	0.002^h^	4.66 ± 0.90	0.481	4.73 ± 0.95	0.256	4.79 ± 0.94	0.355
A' level/diploma	4.72 ± 0.69		4.59 ± 1.03		4.91 ± 0.87		4.53 ± 1.25		4.74 ± 0.93		4.71 ± 0.79		4.69 ± 0.89	
Bachelor	4.58 ± 0.73		4.56 ± 0.85		4.92 ± 0.84		4.01 ± 1.63		4.61 ± 0.86		4.70 ± 0.72		4.64 ± 0.85	
Masters or above	4.43 ± 0.62		4.47 ± 0.75		4.84 ± 0.79		3.77 ± 1.59		4.53 ± 0.77		4.56 ± 0.64		4.52 ± 0.81	
**Residency status**														
Singapore Citizen	4.46 ± 0.67	<0.001^i^	4.46 ± 0.82	0.004^i^	4.84 ± 0.81	0.085	3.97 ± 1.58	0.048	4.50 ± 0.84	<0.001^i^	4.58 ± 0.70	0.001^i^	4.53 ± 0.85	0.002^i^
Permanent Resident	4.61 ± 0.65		4.61 ± 0.83		4.86 ± 0.97		3.76 ± 1.57		4.74 ± 0.79		4.69 ± 0.66		4.67 ± 0.75	
Non Resident	4.84 ± 0.74		4.80 ± 0.88		5.07 ± 0.80		4.38 ± 1.58		4.90 ± 0.76		4.90 ± 0.76		4.90 ± 0.81	
**Position**														
Doctor	4.43 ± 0.63	<0.001^m^^n^	4.55 ± 0.74	<0.001^n^	4.80 ± 0.77	0.193	3.38 ± 1.66	<0.001^m^^n^	4.55 ± 0.72	<0.001^n^	4.56 ± 0.63	<0.001^n^	4.63 ± 0.78	<0.001^n^
Nurse	4.79 ± 0.71		4.72 ± 0.87		4.97 ± 0.87		4.47 ± 1.40		4.83 ± 0.83		4.81 ± 0.76		4.79 ± 0.84	
Allied health	4.36 ± 0.64		4.36 ± 0.81		4.83 ± 0.82		3.75 ± 1.61		4.39 ± 0.82		4.53 ± 0.67		4.43 ± 0.83	
**Years in IMH**														
Less than 1 year	4.61 ± 0.80	0.001^o^^p^	4.54 ± 0.94	0.062	5.11 ± 0.80	0.012^q^	4.12 ± 1.72	0.009^o^	4.65 ± 0.91	0.029^p^	4.77 ± 0.76	0.127	4.66 ± 0.79	0.011^o^
1–5 years	4.51 ± 0.69		4.50 ± 0.84		4.92 ± 0.77		3.95 ± 1.58		4.48 ± 0.84		4.62 ± 0.73		4.57 ± 0.88	
6–10 years	4.35 ± 0.62		4.42 ± 0.76		4.67 ± 0.90		3.69 ± 1.61		4.59 ± 0.86		4.55 ± 0.71		4.43 ± 0.88	
More than 10 years	4.75 ± 0.66		4.72 ± 0.86		4.92 ± 0.87		4.41 ± 1.44		4.78 ± 0.74		4.75 ± 0.65		4.81 ± 0.73	

Significant difference was set at P<0.05 for independent t-test and one-way ANOVA test.

Bonferroni post hot test:

Significant different between:

^a^—Chinese vs Malay;

^b^—Chinese vs Filipino;

^c^—Indian vs Filipino;

^d^—Filipino vs Others;

^e^—Malay vs Indian;

^f^—Malay vs Others;

^g^—Chinese vs Indian;

^h^—Master or above vs Secondary or below & ITE & GCE 'O' level/GCE 'A' level & diploma;

^i^—Singapore Citizen vs Foreigner;

^m^—Nurse vs Doctor;

^n^—Nurse vs Allied Health Staff;

^o^—More than 10 years vs 6–10 years;

^p^—More than 10 years vs 1–5 years;

^q^—6–10 years vs Less than 1 year

Socio-demographic correlates of PMH total and domain specific scores are presented in Table 4. Multiple linear regression analyses revealed that age was significantly correlated with higher spirituality (p = 0.044), PGA (p<0.001), GA (p = 0.002) and PMH total scores (p = 0.01) while for gender, females had higher ES (p<0.001). Significant correlates were also observed across ethnic groups; when compared to Chinese, Malays had significantly higher PMH total, ES and spirituality scores, Indians had higher spirituality, Filipinos had higher PMH total, GC, ES and spirituality scores, while Burmese had higher spirituality. Those who have been married (versus never married) were associated with lower GC (p = 0.014), whereas permanent residents had lower spirituality scores compared to citizens (p = 0.028). When it came to the position held by staff, compared to allied health staff, doctors had lower spiritualty (p = 0.02) and nurses had higher PGA scores (p = 0.021). For years of service at IMH, we found that compared to those who had worked at IMH for more than 10 years, those who had been working at the Institute for 6–10 years had significantly lower PMH total scores (p = 0.018).

**Table 4:** Socio-demographic correlates of PMH total and domain specific scores.

	Total PMH Score				General Coping				Emotional Support				Spirituality				Personal Growth and Autonomy				Interpersonal Skills				Global Affect			p
	β	95% CI		p	β	95% CI		p	β	95% CI		p	β	95% CI		p	β	95% CI		p	β	95% CI		p	β	95% CI		
**Age**	0.011	0.003	0.020	**0.010**	0.009	-0.001	0.019	0.082	0.002	-0.008	0.012	0.738	0.018	0.0005	0.035	**0.044**	0.018	0.008	0.028	**<0.001**	0.008	-0.001	0.017	0.071	0.016	0.006	0.026	**0.002**
**Gender**																												
Male	Ref				Ref				Ref				Ref				Ref				Ref				Ref			
Female	0.066	-0.074	0.205	0.354	-0.053	-0.220	0.115	0.537	0.296	0.128	0.463	**<0.001**	0.105	-0.188	0.398	0.481	-0.042	-0.207	0.124	0.623	0.048	-0.097	0.193	0.516	0.003	-0.163	0.169	0.974
**Ethnicity**																												
Chinese	Ref				Ref				Ref								Ref				Ref				Ref			
Malay	0.309	0.009	0.610	**0.044**	0.224	-0.118	0.566	0.199	0.353	0.016	0.690	**0.040**	1.427	0.843	2.011	**<.0001**	0.059	-0.290	0.408	0.741	0.210	-0.082	0.502	0.159	0.168	-0.174	0.511	0.335
Indian	0.178	-0.036	0.392	0.102	0.060	-0.195	0.315	0.643	0.045	-0.210	0.300	0.730	0.639	0.194	1.084	**0.005**	0.211	-0.045	0.468	0.106	0.216	-0.006	0.438	0.057	-0.010	-0.264	0.245	0.940
Filipino	0.679	0.357	1.000	**<.0001**	0.563	0.187	0.940	**0.003**	0.442	0.057	0.827	**0.025**	2.061	1.406	2.716	**<.0001**	0.184	-0.187	0.555	0.330	0.251	-0.076	0.578	0.132	0.309	-0.066	0.684	0.106
Burmese	0.369	-0.091	0.830	0.115	0.044	-0.456	0.545	0.862	-0.134	-0.646	0.378	0.607	1.203	0.308	2.099	**0.009**	-0.097	-0.627	0.434	0.721	0.037	-0.426	0.501	0.875	0.402	-0.109	0.912	0.123
Others	-0.174	-0.672	0.325	0.493	0.277	-0.289	0.844	0.337	-0.121	-0.721	0.479	0.693	-0.646	-1.639	0.348	0.202	-0.202	-0.790	0.387	0.501	-0.136	-0.679	0.407	0.622	-0.546	-1.111	0.019	0.058
**Marital status**																												
Never married	Ref				Ref				Ref				Ref				Ref				Ref				Ref			
Ever married	-0.048	-0.198	0.103	0.534	-0.222	-0.400	-0.045	**0.014**	0.054	-0.123	0.232	0.546	0.018	-0.291	0.327	0.910	-0.137	-0.316	0.042	0.132	-0.150	-0.303	0.003	0.055	0.055	-0.121	0.230	0.541
**Education level**																												
Secondary/ITE/'O' level	-0.096	-0.484	0.291	0.626	0.053	-0.395	0.500	0.818	-0.321	-0.778	0.136	0.168	0.206	-0.578	0.991	0.606	-0.204	-0.662	0.255	0.383	-0.089	-0.482	0.303	0.655	0.020	-0.434	0.473	0.931
A' level/diploma	0.045	-0.249	0.338	0.765	-0.073	-0.412	0.265	0.671	-0.079	-0.416	0.258	0.646	0.253	-0.338	0.843	0.401	-0.035	-0.380	0.309	0.841	-0.085	-0.385	0.215	0.580	0.085	-0.254	0.424	0.624
Bachelor	-0.049	-0.215	0.117	0.559	-0.082	-0.281	0.118	0.421	-0.057	-0.257	0.143	0.577	-0.241	-0.590	0.108	0.175	-0.074	-0.273	0.124	0.462	-0.016	-0.187	0.156	0.859	0.037	-0.162	0.236	0.716
Masters or above	Ref				Ref				Ref				Ref				Ref				Ref				Ref			
**Residency status**																												
Singapore Citizen	Ref				Ref				Ref				Ref				Ref				Ref				Ref			
Permanent Resident	0.044	-0.187	0.275	0.710	0.076	-0.194	0.347	0.579	0.036	-0.237	0.308	0.796	-0.528	-0.999	-0.057	**0.028**	0.128	-0.146	0.403	0.359	0.051	-0.181	0.284	0.665	0.071	-0.199	0.341	0.606
Non Resident	0.042	-0.229	0.314	0.759	0.096	-0.224	0.415	0.555	0.056	-0.269	0.381	0.734	-0.407	-0.968	0.154	0.154	0.286	-0.034	0.606	0.079	0.185	-0.099	0.469	0.201	0.225	-0.097	0.547	0.170
**Position**																												
Allied health	Ref				Ref				Ref				Ref				Ref				Ref				Ref			
Doctor	-0.064	-0.296	0.167	0.585	0.068	-0.212	0.348	0.635	0.040	-0.240	0.319	0.779	-0.586	-1.077	-0.094	**0.020**	-0.051	-0.329	0.227	0.718	-0.043	-0.285	0.199	0.726	0.056	-0.222	0.334	0.693
Nurse	0.095	-0.107	0.297	0.356	0.128	-0.112	0.368	0.296	0.024	-0.215	0.262	0.846	-0.187	-0.604	0.230	0.379	0.278	0.042	0.515	**0.021**	0.122	-0.087	0.330	0.252	0.055	-0.184	0.294	0.653
**Years in IMH**																												
More than 10 years	Ref				Ref				Ref				Ref				Ref				Ref				Ref			
Less than 1 year	-0.058	-0.359	0.243	0.704	-0.205	-0.562	0.153	0.262	0.135	-0.225	0.494	0.462	-0.097	-0.725	0.531	0.763	0.094	-0.265	0.453	0.606	0.003	-0.306	0.313	0.983	0.078	-0.278	0.435	0.666
1–5 years	-0.079	-0.303	0.145	0.488	-0.127	-0.391	0.138	0.348	0.021	-0.245	0.286	0.879	-0.144	-0.608	0.320	0.542	-0.065	-0.330	0.200	0.631	-0.072	-0.299	0.155	0.534	-0.023	-0.288	0.241	0.862
6–10 years	-0.266	-0.485	-0.046	**0.018**	-0.173	-0.433	0.086	0.191	-0.223	-0.484	0.038	0.093	-0.379	-0.833	0.075	0.101	0.002	-0.257	0.262	0.987	-0.122	-0.347	0.103	0.287	-0.212	-0.471	0.048	0.110
**R Squared**	0.203				0.104				0.091				0.221				0.131				0.088				0.114			

Table 5 summarizes the multivariate linear regression analyses of PMH total scores with job satisfaction and shows that job satisfaction was significantly associated with total PMH for the unadjusted and adjusted models. Compared to the unadjusted model, there was a reduction in the size of association (β = 0.221 to β = 0.184) after the adjustment for socio-demographic variables (model 1), however, the beta coefficient remains almost the same (β = 0.185) after controlling for both socio-demographics and whether the participant has family or friends diagnosed with a mental illness (model 2).

**Table 5:** Unadjusted and adjusted β coefficients (95% CI) representing association of positive mental health with job satisfaction.

	Unadjusted				Model 1^a^				Model 2^b^			
	β	95% CI		p	β	95% CI		p	β	95% CI		p
Job Satisfaction	0.221	0.182	0.260	<.0001	0.184	0.143	0.224	<.0001	0.185	0.145	0.225	<.0001

Unstandardised β coefficient was derived from multivariate linear regression.

^a^ Adjusted for age, gender, ethnicity, marital status, educational level, residency status, position held by the staff, and number of years working at IMH.

^b^ Adjusted for Model 1 plus whether the participant has a family or friend diagnosed with a mental illness.

## Discussion

The findings from this study have shown that there are significant differences in PMH total and domain specific scores across socio-demographic characteristics among doctors, nurses and allied health staff working in a tertiary psychiatric hospital in Singapore. These findings highlight that age and ethnicity were significantly correlated with PMH total scores as well as various domain scores, while gender, marital and residency status and the position held by the staff were only significantly correlated with domain specific scores. Job satisfaction was also found to be a significantly associated with total PMH.

The mean PMH total score was 4.54 while the mean domain specific scores ranged from 4.02 (for spirituality) to 4.89 (for ES). These findings are not unlike to those of community sample in Singapore, where the mean PMH total score was 4.53, and similarly the lowest mean domain score was for spirituality (4.29), while ES had the highest mean score at 4.80 [29]. Among a sample of outpatients with affective disorders, the mean PMH total score was lower (3.74), however among this sample the lowest mean domain score was for GA (3.31) whilst IS was the highest (4.21) [30].

Age was significantly associated with PMH total, spirituality, PGA and GA scores, where, as age increased, so did the respective scores. It is important to highlight however that the beta scores for each of these domains were very small, suggesting a slight but still significant increase. These findings resonate with that of previous research where in old age, well-being and positive affect increased [31,32]. Our study found that older staff have higher spirituality which is not dissimilar to findings in local and Western settings [29,33]. Various explanations have been proposed; firstly for spirituality, it is believed that higher stages of faith are characterized by a sense of unity, transcendence and wisdom that can be achieved with age [34,35], while Moxey et al., [36] related the higher spirituality among older individuals with the physical strains experienced with age and propose its role as a coping mechanism. With regards to PGA, our findings conflict those of previous research, whereby PGA scores actually increased with age. Cross-sectional and longitudinal studies have consistently shown however that personal growth declines with age [37,38]. As the majority of our sample was aged below 40 years, further research exploring the effects of age on PGA as well as other PMH domains among older health professionals is warranted.

Ethnicity was a strong predictor of total PMH as well as the GC, ES and spirituality domains, with Malays, Indians, Filipinos and Burmese scoring significantly higher across one or more of these domains when compared to Chinese. Local studies have found similar results where Chinese consistently score lower than Malays and Indians [29,30] for spirituality. Malays often have greater religiosity (assessed by frequency of prayers and attendance at religious services) compared to Chinese and Indians, and most Malays and Indians consider religion important to their daily life compared to Chinese [39]. With regards to ES, Malays and Filipinos had significantly higher scores than Chinese. Culture and emotions have been extensively studied, with ethnographic studies dating back to the 1930’s, which found that emotional expression among the Chinese, whilst at times is similar to that of Western cultures, can also differ, whereby they emphasize social congruence over individual expression [40]. Potter [41] proposed that emotions lack social significance in the collectivistic Chinese culture and thus are less relevant than in individualistic culture, where for the Chinese, emotional expression is not so much discouraged or suppressed, but rather it is ignored. Potter’s findings support the earlier work of Klineberg, highlighting low levels of emotional experience and sparse emotional displays among Chinese, which may explain the ethnic differences observed in the current study.

Compared to Chinese participants, Filipinos scored higher for total PMH, GC, ES and spirituality domains, suggesting there are some inherent cultural differences between the two ethnic groups. Connor [42] explored the cultural influences on coping strategies amongst Filipino nurses working in USA and found six themes relating to coping behaviours and strategies: familial coping, intra-cultural coping, fate and faith based coping, forbearance (patience and self-control) and contentment, affirming the nursing profession and proving themselves, and escape and avoidance and it is possible that these cultural influences explain the findings in our study. Given Filipinos commonly use faith-based coping strategies, it is not surprising they also scored highly on the spirituality domain. Religious practice has been shown to augment social support and coping skills in the face of adversity, whilst providing a buffer against stressful life events [43,44] and this ‘buffering’ or inter-relationship between various aspects of PMH may explain why spirituality along with other domains such as GC and ES were also higher amongst certain ethnic groups.

The effects of gender, marital and residency status and education on PMH and its domains were minimal. The only significant gender difference observed for both bivariate and multivariate analysis was for the ES domain, where females had higher ES compared to their male counterparts. Gross and John [45] hypothesised that men and women differ in their strategies to regulate emotions and these are mediated by ‘reappraisal’ and ‘suppression’ which influence their affect, well-being and social relationships. Similarly, marital status was only a predictor of one PMH domain, GC, where participants who have been married displayed lower scores compared to those who were single (never married). Whilst the benefits of marriage are well documented in terms of sizeable physical and psychological well-being advantages [46], many of these benefits are explained via the social support [47] associated with marriage, although this was not a significant predictor of ES in the current study. It is possible that singles, as a result of not having marital social support, are better able to cope on their own however this theory requires further exploration.

Interestingly, we found that permanent residents had lower spirituality when compared to Singapore citizens. This finding may be a result of the small number of permanent residents among this sample (12.8%), who were predominantly younger in age and given that spirituality was higher among older age participants, this may be a possible explanation for this finding. We did not observe any differences by education level and this is most likely due to our sample comprising only of mental health professionals who were highly educated, with the majority obtaining at least a tertiary qualification and therefore distinctions could not be made.

Employment related correlates showed that compared to allied health staff, doctors had significantly lower spirituality scores, while nurses had higher PGA scores. We did not ask respondents about their religion or religious beliefs and therefore it is difficult to postulate why doctors scored lower on the spirituality domain. Upon further analysis we did however find that Chinese doctors scored lower on spirituality compared to nurses and allied health staff of other ethnicities and this may explain the finding. Regarding nurses having higher PGA scores compared to allied health staff, this could be a result of the autonomy and skills required in their profession, which is often regarded as physically and emotionally demanding, although the specific PGA questions are more general and not specific to their job or role. Finally, those working at IMH for 6–10 years had lower PMH total scores compared to staff who had worked at the hospital for more than 10 years. This could be a result of job satisfaction, career development and progression, or staff’s attitudes towards their job and their patients. Findings from an earlier study among nurses working at the same psychiatric hospital revealed that nurses with more than 10 years of psychiatric nursing experience had more positive attitudes towards those with a mental illness [48] and this may be an underlying factor contributing to their overall higher PMH.

As expected, job satisfaction strongly correlated with higher PMH and this positive relationship has consistently been reported among nurses and clinicians [49,50]. Job satisfaction directly impacts individual’s health and well-being and has been associated with physical symptoms [51], emotional states and anxiety [52], and depression [53]. More specifically to healthcare, job satisfaction can also affect productivity, performance, quality of care and patient safety [54]. Ryan and Deci’s self-determination theory postulates that work environments that provide competence, autonomy, and relatedness (three intrinsic psychological needs), promote work engagement, motivation and psychological well-being [55,56]. Research has also shown that workplace satisfaction with these three needs is linked to various performance, attitudinal and well-being related outcomes [57–59] and further emphasizes the importance of supportive and conducive workplace settings to overall PMH and well-being.

The study findings should be viewed in light of the following limitations. This was a cross-sectional study among staff working at IMH and therefore these findings are not generalizable to all IMH staff or the overall Singapore population, nor could causal relationships be established. The study was limited to doctors, nurses and allied health staff and therefore the PMH of other staff including health care attendants, patient services associates and administrative staff may differ. The multi-dimensional PMH instrument used in this study was locally developed and validated among residents aged 21–65 years old belonging to the three main ethnic groups in Singapore- Chinese, Malays and Indians [14]. However the current study included participants up to the age of 70 and other ethnic groups including Filipinos and Burmese. It is therefore possible that other dimensions of PMH among these staff were not captured. The PMH instrument and other data collected were based on self-report and therefore respondents may have provided socially desirable responses. Whilst socio-demographic information was captured within the survey, data pertaining to salary was omitted, due to concerns raised during the ethical review process, whereby it was highlighted that staff may not be willing to provide such personal information. Finally, whilst the purpose of the current study was to explore PMH amongst mental health professionals, using a locally developed and validated instrument, the concurrent use of more widely used outcome measures such as the Maslach Burnout Inventory could further strengthen the study and warrants exploration in the future.

These limitations notwithstanding, this is the first study to explore PMH among staff working at a tertiary psychiatric hospital in Singapore and has highlighted important differences in PMH by socio-demographic characteristics, and the important correlation between PMH and job satisfaction. These findings have important implications: they emphasize the importance of PMH to individuals, communities and society as a whole. In order to promote and foster PMH, workplaces need to consider the importance of psychosocial well-being and the mental health wellness of staff whilst providing policies, facilities, and an environment that supports and maintains overall health and work efficiency.

Although extensive research has explored the causes and correlates of well-being, very little has focused on ways in which well-being can be reliably enhanced [60]. More recently, there has been a focus on utilizing an individual’s strengths in order to facilitate well-being. Seligman et al., [61] in their review of the effectiveness of positive psychology interventions found that increased well-being was observed among individuals who were instructed to develop their character strengths. Similarly, studies have also shown strong links between strengths-based development and employee engagement, whereby they are analogous to the concept of employee well-being [62].

The workplace is a key environment that affects the mental health and well-being of working adults [63] and therefore interventions to enhance well-being such as utilizing strengths are recommended. In addition, effective approaches to mental health promotion in the workplace which address key influencing factors such as social support, enhanced job control, increased staff involvement, workload assessment, effort/reward balance, role clarity and policies to tackle bullying and harassment [64,65] are also equally important to employee well-being, job satisfaction and total PMH. Key components of PMH such as GC and PGA are particularly important in the workplace as staff gain more tasks and responsibilities and therefore providing opportunities to build and strengthen these attributes or skills would be beneficial to individual staff and organisations as a whole.

## Supporting information

S1 Table PMH total and domain scores by allied health discipline. (DOCX)

## Abbreviations

ANOVA: Analysis of variance
CFI: Comparative fit indexes
ES: emotional support
GA: global affect
GC: general coping
IMH: Institute of Mental Health
IS: interpersonal skills
PGA: personal growth and autonomy
PMH: positive mental health
RMSEA: Root Mean Square Error Approximation
TLI: Tucker-Lewis Index
WHO: World Health Organization

## References

[1] World Health Organization (2001). World health report—Mental Health: New Understanding, New Hope.

[2] World Health Organization (2005) Mental Health Action Plan for Europe: Facing the Challenges, Building Solutions. Geneva, Switzerland: World Health Organization.

[3] Prince M, Patel V, Saxena S, Maj M, Maselko J, Phillips MR et al (2007). No health without mental health. Lancet 370, 859–877. 10.1016/S0140-6736(07)61238-0 17804063

[4] Ryan RM, Deci EL. (2001) On happiness and human potentials: a review of research on hedonic and eudaimonic well-being. Annual Review of Psychology 52,141–166. 10.1146/annurev.psych.52.1.141 11148302

[5] Waterman AS (1993) Two conceptions of happiness: contrasts of personal expressiveness (eudaimonia) and hedonic enjoyment. Journal of Personality and Social Psychology, 64, 678–691.

[6] Merrill RM, Aldana SG, Pope JE, Anderson DR, Coberley CR, Vyhlidal TP, et al (2011) Evaluation of a Best-Practice Worksite Wellness Program in a Small-Employer Setting Using Selected Well-being Indices. Journal of Occupational and Environmental Medicine, 53:448–454. 10.1097/JOM.0b013e3182143ed0 21407092

[7] Ford E, Clark C, Stansfeld SA (2011) The influence of childhood adversity on social relations and mental health at mid-life. Journal of Affective Disorders, 133:320–7. 10.1016/j.jad.2011.03.017 21482436

[8] Lehman AF (1983) The Well-being of Chronic Mental Patients: Assessing their quality of life. Archives of General Psychiatry, 40(4):369–373. 6838316 10.1001/archpsyc.1983.01790040023003

[9] Sorrell JM (2011) Mental Health of the Oldest-Old. Journal of Psychosocial Nursing and Mental Health Services, 13:1–3. 10.3928/02793695-20110329-04 21485976

[10] Rogers A & Pilgrim D (2005) A Sociology of Mental Health and Illness. Maidenhead, UK: Open University Press.

[11] Keyes CL. (2002) The mental health continuum: from languishing to flourishing in life. Journal of Health and Social Behaviour, 43,207–222. 12096700

[12] Vaingankar JA, Subramaniam M, Lim YW, Sherbourne C, Luo N, Ryan G, et al (2012) From well-being to positive mental health: Conceptualization and qualitative development of an instrument in Singapore. Quality of Life Research; 21:1785–94. 10.1007/s11136-011-0105-3 22286222

[13] Statistics of Singapore (2015). Accessed on 27 May 2016. Accessed at: https://www.singstat.gov.sg/docs/default-source/default-document-library/publications/publications_and_papers/population_and_population_structure/population2015.pdf

[14] Vaingankar JA, Subramaniam M, Chong SA, Abdin E, Edelen MO, Picco L, et al (2011). The Positive Mental Health Instrument: Development and validation of a culturally relevant scale in a multi-ethnic Asian population. Health and Quality of Life Outcomes; 9:92 10.1186/1477-7525-9-92 22040157 PMC3229450

[15] Vaingankar JA, Abdin E, Chong SA, Sambasivam R, Jeyagurunathan A, Seow E, et al (2016). Psychometric properties of the positive mental health instrument among people with mental disorders: a cross-sectional study. Health Quality of Life Outcomes, 12;14:19 10.1186/s12955-016-0424-8 26868835 PMC4751680

[16] Page KM and Vella-Brodrick DA (2009). The ‘What’, ‘Why’ and ‘How’ of Employee Well-Being: A New Model Social Indicators Research; 90:441–458

[17] Wright T. A., & Bonett D. G. (2007). Job satisfaction and psychological well-being as nonadditive predictors of workplace turnover. Journal of Management, 33, 141–160.

[18] Wright T. A., & Cropanzano R. (2000). Psychological well-being and job satisfaction as predictors of job performance. Journal of Occupational Health Psychology, 5, 84–94. 10658888 10.1037//1076-8998.5.1.84

[19] Kanste O. (2011). Work engagement, work commitment and their association with wellbeing in health care. Scandinavian Journal of Caring Sciences, 25 (4), 754–761 10.1111/j.1471-6712.2011.00888.x 21564150

[20] Sun T., Zhao X.W., Yang L.B., Fan L.H. (2012). The impact of psychological capital on job embeddedness and job performance among nurses: a structural equation approach. Journal of Advanced Nursing, 68 (1), 69–79. 10.1111/j.1365-2648.2011.05715.x 21645045

[21] Volpe U, Luciano M, Palumbo C, Sampogna G, Del Vecchio V, Fiorillo A. (2014). Risk of burnout among early career mental health professionals. Journal of Psychiatric and Mental Health Nursing. 21, 774–81. 10.1111/jpm.12137 25757038

[22] Madathil R, Heck NC, Schuldberg D (2014) Burnout in psychiatric nursing: Examining the interplay of autonomy, leadership style and depressive symptoms. Archives of Psychiatric Nursing 28,160–166. 10.1016/j.apnu.2014.01.002 24856267

[23] Yang S, Meredith P, Khan A. (2015) Stress and burnout among healthcare professionals working in a mental health setting in Singapore. Asian Journal of Psychiatry 15:15–20 10.1016/j.ajp.2015.04.005 25922279

[24] Sanchez-Reilly S., Morrison L.J., Carey E., Bernacki R., O’neill L., Kapo J., et al (2013). Caring for oneself to care for others: physicians and their self-care. Journal of Supportive Oncology. 11, 75–81. 23967495 10.12788/j.suponc.0003 PMC3974630

[25] Scanlan J.N., Still M., (2013). Job satisfaction, burnout and turnover intention in occupational therapists working in mental health. Australian Occupational Therapy Journal, 60, 310–318. 10.1111/1440-1630.12067 24089982

[26] Edwards D., Burnard P (2003). A systematic review of stress and stress management interventions for mental health nurses. Journal of Advanced Nursing, 42, 169–200. 12670386 10.1046/j.1365-2648.2003.02600.x

[27] Stafford-Brown J., Pakenham K.I. (2012). The effectiveness of an ACT informed intervention for managing stress and improving therapist qualities in clinical psychology trainees. Journal of Clinical Psychology, 68, 513–592. 10.1002/jclp.21844 22566279

[28] Letvak S, Ruhm CJ, McCoy T. (2012) Depression in Hospital Employed Nurses. Clinical Nurse Specialist, 26(3), 177–82. 10.1097/NUR.0b013e3182503ef0 22504476

[29] Vaingankar JA, Subramaniam M, Abdin E, Picco L, Phua A, Chua BY, et al (2013) Socio-demographic Correlates of Positive Mental Health and Differences by Depression and Anxiety in an Asian Community Sample. Annals Academy of Medicine Singapore, 42(10):514–23. 24254238

[30] Seow LSE, Vaingankar JA, Abdin E, Sambasivam R, Jeyagurunathan A, Pang S, et al (2016). Positive mental health in outpatients with affective disorders: Associations with life satisfaction and general functioning Journal of Affective Disorder;190:499–507 10.1016/j.jad.2015.10.021 26561940

[31] Carstensen LL. (1998). A life-span approach to social motivation In Motivation and Self-Regulation Across the Life Span, ed. Heckhausen J, Dweck CS, pp. 341–64. New York: Cambridge Univ. Press

[32] Mroczek DK, Kolarz CM. (1998). The effect of age on positive and negative affect: a developmental perspective on happiness. Journal of Personality and Social Psychology. 75:1333–49 9866191 10.1037//0022-3514.75.5.1333

[33] Westerhof GJ and Keyes CLM (2010). Mental Illness and Mental Health: The Two Continua Model Across the Lifespan. Journal of Adult Development,17:110–119 10.1007/s10804-009-9082-y 20502508 PMC2866965

[34] Fowler J. Stages of faith. New York: Harper & Row, 1981.

[35] Wink P, Dillon M (2002). Spiritual development across the adult life course: Findings from a longitudinal study. Journal of Adult Development; 9:79–94.

[36] Moxey A, McEvoy M, Bowe S, Attia J (2011). Spirituality, religion, social support and health among older Australian adults. Australasian Journal of Ageing; 30;82–88. 10.1111/j.1741-6612.2010.00453.x 21672117

[37] Springer KW, Pudrovska T and Hauser RM. (2011) Does psychological well-being change with age? Longitudinal tests of age variations and further exploration of the multidimensionality of Ryff’s model of psychological well-being. Social Science Research 40: 392–398 10.1016/j.ssresearch.2010.05.008 21218154 PMC3014614

[38] Ryff Carol D.; Keyes Corey LM.; Hughes Diane L. (2003) Status Inequalities, Perceived Discrimination, and Eudaimonic Well-Being: Do the Challenges of Minority Life Hone Purpose and Growth? Journal of Health and Social Behavior; 44:275–291 14582308

[39] Institute of Southeast Asian Studies, Abu Bakar Noor Laily binti Dato’ (1985). Ethnicity and fertility in Malaysia. Singapore: Institute of Southeast Asian Studies.

[40] Klineberg O. (1938). Emotional expression in Chinese literature. Journal of Abnormal and Social Psychology, 33, 517–520.

[41] Potter S. H. (1988). The cultural construction of emotion in rural Chinese social life. Ethos, 16, 181–208.

[42] Connor JB. (2016) Cultural Influence on Coping Strategies of Filipino Immigrant Nurses, Workplace Health & Safety, 64;5:195–201 27026273 10.1177/2165079916630553

[43] Grosse-Holforth M., Pathak A., Koenig H. G., Cohen H. J., Pieper C. F., & Vanhook L. G. (1996). Medical illness, religion, health control and depression of institutionalized medically ill veterans in long-term care. International Journal of Geriatric Psychiatric, 2, 613–620

[44] Tix A. P., & Frazier P. A. (1998). The use of religious coping during stressful life events: Main effects, moderation, and mediation. Journal of Consulting and Clinical Psychology, 66(2), 411–422. 9583344 10.1037//0022-006x.66.2.411

[45] Gross JJ, John OP (2003). Individual differences in two emotion regulation processes: Implications for affect, relationships, and well-being. Journal of Personal and Social Psychology; 85:348–62. 10.1037/0022-3514.85.2.348 12916575

[46] Shapiro A, Keyes CLM (2008). Marital Status and Social Well-Being: Are the Married Always Better Off? Social Indicators Research 88:329–346

[47] House J. S., Landis K. R., & Umberson D. (1988). Social relationships and health. Science, 241, S^#0–S45. 10.1126/science.3399889 3399889

[48] Tay SEC, Pariyasami S, Ravindran K, Ali MI, Rowsudeen MT (2004) Nurses’ attitudes toward people with mental illnesses in a psychiatric hospital in Singapore. Journal of Psychological Nursing, 42;10: 41–47 10.3928/02793695-20041001-08 15543671

[49] Olatunde BE, Odusanya O (2015). Job Satisfaction and Psychological wellbeing among mental Health Nurses. International Journal of Nursing Didactics, 5: 8

[50] Healy C, Mckay M. (2000) Nursing stress, the effects of coping strategies and job satisfaction in a sample of Australian nurses. Journal of Advanced Nursing; 31(3): 681–8 10718888 10.1046/j.1365-2648.2000.01323.x

[51] Begley P, Czajka M. (1993) Relationship between Job satisfaction and Organizational Commitment. Journal of American Science, 3: 45–60.

[52] Jex R, Gudanowski T (2010). Job satisfaction among Nurses in general practice. British Nursing Journal; 23(4): 567–75

[53] Barlings T, Burns U (2009) Factors affecting retention and turn over among Nurses in America. American Journal of Nursing, 43(4): 345–54

[54] Murrelis T, Robinson S, Griffiths P. (2008) Job Satisfaction trends during nurses early career. Biomedical Journal, 34: 37–45 10.1186/1472-6955-7-7 PMC2435528 18534023

[55] Deci E. L., & Ryan R. M. (1985). Intrinsic motivation and self-determination in human behavior. New York, NY: Plenum.

[56] Ryan R. M., & Deci E. L. (2000). Self-determination theory and the facilitation of intrinsic motivation, social development, and well-being. American Psychologist, 55, 68–78. 11392867 10.1037//0003-066x.55.1.68

[57] Baard P. P., Deci E. L., Ryan R. M. (2004). Intrinsic need satisfaction: A motivational basis of performance and well-being in two work settings. Journal of Applied Social Psychology, 34, 2045–2068.

[58] Deci E. L., Ryan R. M., Gagné M., Leone D. R., Usunov J., & Kornazheva B. P. (2001). Need satisfaction, motivation, and well-being in the work organizations of a former Eastern Bloc country. Personality and Social Psychology Bulletin, 27, 930–942

[59] Ilardi B. C., Leone D., Kasser R., & Ryan R. M. (1993). Employee and supervisor ratings of motivation: Main effects and discrepancies associated with job satisfaction and adjustment in a factory setting. Journal of Applied Social Psychology, 23, 1789–1805.

[60] Sheldon K. M., & Lyubomirsky S. (2006). Achieving sustainable gains in happiness: Change your actions not your circumstances. Journal of Happiness Studies, 7, 55–86.

[61] Seligman M. E. P., Steen T. A., Park N., & Peterson C. (2005). Positive Psychology progress: Empirical validation of interventions. American Psychologist, 60, 410–421. 10.1037/0003-066X.60.5.410 16045394

[62] Harter J. K., Schmidt F. L., & Keyes C. L. M. (2003). Well-being in the workplace and its relationship to business outcomes: A review of the Gallup studies In Keyes C. L. M. & Haidt J. (Eds.), Flourishing: The positive person and the good life. Washington, DC: American Psychological Society.

[63] World Health Organisation (2000). Mental health and work: Impact, issues and good practices

[64] van der Klink JJ, Blonk RW, Schene AH, van Dijk FJ (2001). The benefits of interventions for work-related stress. American Journal of Public Health;91(2):270–6. 11211637 10.2105/ajph.91.2.270 PMC1446543

[65] Michie S, Williams S (2003). Reducing work related psychological ill health and sickness absence: a systematic literature review. Occupational and Environmental Medicine. 1;60(1):3–9. 10.1136/oem.60.1.3 12499449 PMC1740370

