# Supplementary material for: Positive mental health among health professionals working at a psychiatric hospital
Source: PLoS One. 2017 Jun 7;12(6):e0178359. doi: 10.1371/journal.pone.0178359 (PMC5462373; doi:10.1371/journal.pone.0178359)
Supplement: S1 Table — (DOCX) [file pone.0178359.s001.docx]

**S1 Table: PMH total and domain scores by allied health discipline**

|  |  | Total PMH Score | | General Coping | | Emotional Support | | Spirituality | | Personal Growth and Autonomy | | Interpersonal Skills | | Global Effects | |
| --- | --- | --- | --- | --- | --- | --- | --- | --- | --- | --- | --- | --- | --- | --- | --- |
|  | N | Mean | SD | Mean | SD | Mean | SD | Mean | SD | Mean | SD | Mean | SD | Mean | SD |
| Psychologist | 43 | 4.316 | 0.528 | 4.310 | 0.687 | 4.827 | 0.718 | 3.541 | 1.440 | 4.528 | 0.721 | 4.538 | 0.630 | 4.279 | 0.913 |
| Pharmacist | 28 | 4.120 | 0.737 | 4.302 | 0.961 | 4.593 | 0.889 | 2.867 | 1.655 | 4.011 | 0.935 | 4.440 | 0.710 | 4.473 | 0.750 |
| Occupational therapist | 25 | 4.302 | 0.556 | 4.218 | 0.888 | 4.810 | 0.745 | 3.949 | 1.645 | 4.244 | 0.713 | 4.431 | 0.704 | 4.250 | 0.594 |
| Physiotherapist | 3 | 5.080 | 0.433 | 4.704 | 0.501 | 5.286 | 1.010 | 4.429 | 0.515 | 5.233 | 0.577 | 4.667 | 0.676 | 5.917 | 0.144 |
| Medical Social worker | 47 | 4.434 | 0.628 | 4.390 | 0.666 | 4.897 | 0.730 | 4.246 | 1.549 | 4.338 | 0.703 | 4.461 | 0.658 | 4.337 | 0.855 |
| Case Manager | 57 | 4.471 | 0.688 | 4.458 | 0.903 | 4.891 | 0.947 | 3.818 | 1.639 | 4.533 | 0.897 | 4.663 | 0.670 | 4.609 | 0.809 |
